# Tweet Topics and Sentiments Relating to COVID-19 Vaccination Among Australian Twitter Users: Machine Learning Analysis

**DOI:** 10.2196/26953

**Published:** 2021-05-19

**Authors:** Stephen Wai Hang Kwok, Sai Kumar Vadde, Guanjin Wang

**Affiliations:** 1 School of Nursing The Hong Kong Polytechnic University Hong Kong Hong Kong; 2 Discipline of Information Technology, Media and Communications Murdoch University Perth Australia

**Keywords:** COVID-19, vaccination, public topics, public sentiments, Twitter, infodemiology, infoveillance, social listening, infodemic, social media, natural language processing, machine learning, latent Dirichlet allocation

## Abstract

**Background:**

COVID-19 is one of the greatest threats to human beings in terms of health care, economy, and society in recent history. Up to this moment, there have been no signs of remission, and there is no proven effective cure. Vaccination is the primary biomedical preventive measure against the novel coronavirus. However, public bias or sentiments, as reflected on social media, may have a significant impact on the progression toward achieving herd immunity.

**Objective:**

This study aimed to use machine learning methods to extract topics and sentiments relating to COVID-19 vaccination on Twitter.

**Methods:**

We collected 31,100 English tweets containing COVID-19 vaccine–related keywords between January and October 2020 from Australian Twitter users. Specifically, we analyzed tweets by visualizing high-frequency word clouds and correlations between word tokens. We built a latent Dirichlet allocation (LDA) topic model to identify commonly discussed topics in a large sample of tweets. We also performed sentiment analysis to understand the overall sentiments and emotions related to COVID-19 vaccination in Australia.

**Results:**

Our analysis identified 3 LDA topics: (1) attitudes toward COVID-19 and its vaccination, (2) advocating infection control measures against COVID-19, and (3) misconceptions and complaints about COVID-19 control. Nearly two-thirds of the sentiments of all tweets expressed a positive public opinion about the COVID-19 vaccine; around one-third were negative. Among the 8 basic emotions, trust and anticipation were the two prominent positive emotions observed in the tweets, while fear was the top negative emotion.

**Conclusions:**

Our findings indicate that some Twitter users in Australia supported infection control measures against COVID-19 and refuted misinformation. However, those who underestimated the risks and severity of COVID-19 may have rationalized their position on COVID-19 vaccination with conspiracy theories. We also noticed that the level of positive sentiment among the public may not be sufficient to increase vaccination coverage to a level high enough to achieve vaccination-induced herd immunity. Governments should explore public opinion and sentiments toward COVID-19 and COVID-19 vaccination, and implement an effective vaccination promotion scheme in addition to supporting the development and clinical administration of COVID-19 vaccines.

## Introduction

### The COVID-19 Pandemic

COVID-19 is an infectious disease caused by the novel coronavirus SARS-CoV-2, which was first identified in Wuhan, China, in December 2019 [1]. As of early January 2021, the cumulative number of confirmed cases was 83,862,300, while the number of deaths was 1,837,253, affecting 222 countries or regions globally [2]. In Australia, the total number of confirmed cases was 28,483, and the number of deaths was 909 in early January 2021 [3]. Both the incidence and prevalence have been rising globally, although these rates differ across countries [4]. In 2020, the pandemic had significant negative impacts on individuals, governments, and the global economy [5,6].

Patients with COVID-19 could experience either no symptoms, common signs and symptoms of infection, or respiratory distress, or die from the disease. The proportion of asymptomatic patients was estimated at 16%, with the proportion in children being nearly double that of adults [7,8]. However, over 80% of those who were asymptomatic had either unilateral or bilateral pulmonary involvement in computerized tomography scans [8]. Among those who were symptomatic, fever, cough, and fatigue were the most common symptoms [9,10]. Five percent of patients with COVID-19 developed acute respiratory distress syndrome [11]. Among them, the death rate ranged between 13% and 69% across countries [12].

The virus could be transmitted through close contact, or even droplets, between individuals, where the mucous membranes of healthy individuals are exposed to secretions produced by the carriers [13]. The reproductive number (R_0_) of COVID-19 was approximately 3 but varies from 2 to 7 across countries [14,15]. This means one carrier could infect 3 individuals on average. Under public infection control measures, social distancing does not seem applicable to family households where the risk of transmission is high. A meta-analysis of 24 studies found that the intrafamily transmission rate of SARS-CoV-2 was higher than the transmission rate of severe acute respiratory syndrome coronavirus (SARS-CoV) or Middle East respiratory syndrome coronavirus (MERS-CoV) in households [16], which may contain vulnerable groups such as the elderly, those who are immunocompromised, or have chronic diseases.

### Background on Vaccination

Briefly, the purpose of vaccination is to allow the immune system to memorize the features of the targeted pathogen and be able to initiate an immune response that is fast and strong enough to defeat the live pathogen in the future. Over 115 vaccines for COVID-19 are undergoing investigation and trials, and most of them target the spike protein of SARS-CoV-2 [17]. The development of a vaccine usually takes years. The relatively fast development of the COVID-19 vaccine could be ascribed to previous work on vaccines for SARS-CoV, which is 80% similar to SARS-CoV-2, as well as the immense and urgent need for vaccination [18].

Vaccination that is evidence-based and officially approved by health authorities is generally safe. The adverse effects, as well as their incidence rates, vary across types of vaccines. Previous studies have reported the incidence rates of severe adverse reactions in general populations after receiving vaccines. For example, the incident rate of febrile seizures after receiving the measles, mumps, and rubella (MMR) and varicella vaccine was 8.5 per 10,000 doses [19]. The rates attributable to influenza vaccines or 13-valent pneumococcal conjugate vaccines (PCV13) were 13 to 45 per 100,000 doses [20]. On the other hand, the incident rate of thrombocytopenic purpura after MMR injection was 1 per 20,000 doses [19]. Moreover, the incidence rates of some rare diseases such as intussusception after rotavirus vaccine injection ranged from 1 to 5 per 100,000 doses [20]. There was insufficient evidence to conclude that vaccination was the direct cause of the severe adverse effects compared with the vast majority of those who benefited from vaccinations.

Vaccination is a collective strategy that needs a high proportion of the population to be vaccinated in order to generate a protective effect. The proportion is calculated as (R_0_–1)/R_0_ [21]. If one patient could infect 3 individuals, then the proportion of the population that needs to be vaccinated would be two-thirds. This two-thirds should comprise individuals who have normally functioning immune systems. Those who are immunocompromised are contraindicated to certain types of vaccines such as live vaccines because of poor responses or severe adverse reactions [22,23]. Severe allergic reaction to a vaccine is a contraindication, although the risk is as small as 1 per 1,000,000 doses [19]. Hence, the higher the proportion of those who have normal immune systems receiving vaccinations, the better for achieving herd immunity to protect oneself and others.

### Exploring Public Opinion on the COVID-19 Vaccine

In the last two decades, a prominent antivaccination movement has risen, resulting in a decline in MMR vaccination coverage and a rise in measles outbreaks in the United States, the United Kingdom, and certain major European countries [24]. A case study, which proposed an association between the MMR vaccine and autism [25], although disproven by several studies in subsequent years [26-31], fueled the antivaccine movement, and then was retracted [32]. Nevertheless, the adverse factors promoting antivaccination might be ignoring high-level evidence such as the results of randomized controlled trials of vaccines [33-35] as well as a selective adoption of unverified information by the public.

Social media has become a frequently used platform to disseminate both authorized information and misinformation. Authorized sources such as the World Health Organization [36], the US Centers for Disease Control and Prevention [37], the US Food and Drug Administration [38], and the UK Department of Health and Social Care [39] are available online. However, previous studies showed that around 30% to 60% of the information related to vaccination on social media were antivaccine content [24]. In websites that provided vaccine-related information, over 50% contained inaccurate information [40]. Although antivaxxers proposed different rationales to oppose vaccination [41], the fact is that only vaccination has a history of successfully eradicating viral diseases such as smallpox [42].

As several COVID-19 vaccine trials are progressing to or have nearly completed phase 3 in the second half of 2020, it is expected that vaccines will be made available to the public by 2021 [43,44]. In Australia, Dodd et al [45] conducted an online survey of 4362 adults in mid-April 2020 about 1 month after lockdown measures had been imposed. They found that 86% of the sample claimed that they would get the COVID-19 vaccine when available. At that time, 65% to 75% of the respondents were confident in the federal and state governments’ responses. On August 19, the Australian prime minister [46] announced that the government had made an agreement with AstraZeneca: if its COVID-19 vaccine is proven to be safe and effective, Australia could manufacture it and make it free for the public. Later, the University of Oxford and AstraZeneca [47] and Johnson & Johnson [48] paused their vaccine trials in mid-September and mid-October 2020, respectively, to investigate adverse reactions among participants during the trials, which were resumed after investigations.

Significant health care–related events, such as news about vaccine efficacy [49], disease outbreak [50], or legislative decree of mandatory vaccinations [51], were found to trigger public discussions on social media. However, negative news about the vaccine, as well as antivaccine sentiment, could be hurdles to achieving vaccination-induced herd immunity. For example, information associated with the adverse effects of vaccinations were commonly manipulated by antivaxxers to fuel their movements [52]. They had even started using conspiracy theories against developing COVID-19 vaccines even before development had begun [53-55]. Therefore, online public opinion and sentiments around COVID-19 vaccination need to be explored and reviewed to promote public vaccination schemes based on factors affecting vaccination acceptance.

This study aimed to explore major topics and sentiments of tweets about COVID-19 vaccination among Twitter users in Australia. Findings from this study could help governments and health agencies plan, modify, and implement a timely promotion of vaccination to achieve vaccination-induced herd immunity.

## Methods

### Data Collection

Twitter, one of the world’s major social media platforms, with 187 million daily active users as of the third quarter of 2020 [56], was chosen as the data source. Twitter is a common source of text for sentiment analysis [57,58] and analysis of sentiments toward vaccinations [59,60]. We used the R library package *rtweet* [61] to access the Twitter premium API (application programming interface) service and collect COVID-19 vaccine–related tweets posted between January 22 and October 20, 2020. Retweets, non-English tweets, and tweets with a geolocation outside Australia were excluded. The search terms “vacc OR vax OR vaccine OR vaccination” AND “corona OR covid” were used to search target tweets. Boolean operators “AND” and “OR” guaranteed that tweets that contained words belonging to the root of “vaccine” as well as the root of either “coronavirus” or “COVID” could be searched. As a result, 31,100 tweets were collected and used in this study. The number of tweets collected from January 22 to October 20, 2020, are shown in Multimedia Appendix 1.

### Data Preprocessing

The R library packages of *qdapRegex* [62] and *tm* [63,64] were used for the preprocessing of text. The procedures included (1) removal of non-English words or common words that do not provide insights into a specific topic (eg, stop words); (2) case folding, which changes words into lower case for stemming; and (3) stemming of inflected words into roots, followed by stem completion to return complete words (tokens) for the results visualizations. The custom stop words removed were “amp” (ampersands) and the inflected words derived from “vaccine,” “coronavirus,” and “COVID.” In addition to that, all stop words with reference to those in the package *tm*, Python libraries *spaCy* [65] and *gensim* [66], as well as stop words suggested by Sedgewick and Wayne [67] and the SAO/NASA (Smithsonian Astrophysical Observatory/National Aeronautics and Space Administration) Astrophysics Data System [68], were also removed in the corpus. Stop words in Python libraries and in other aforementioned sources were extracted and assigned to an R object for the ease of process in R. In addition, the dictionary used for stem completion was a corpus saved before the stemming procedure.

### Associations Between Word Tokens

The word tokens were sorted by their counts in the corpus and plotted against their counts as shown in Multimedia Appendix 2. It was observed that the inflection point of the concave-up, decreasing curve was located at approximately 250 counts. Thus, word tokens having counts greater than 250 were included in pairwise correlation tests. The R library package *widyr* [69] was used to compute the correlations between word tokens. Then, the word pairs with Pearson correlation coefficients larger than 0.1 were plotted in a network graph. Coefficients smaller than 0.1 were considered negligible [70,71]. On the other hand, word pairs were also sorted by their counts and plotted against the counts as shown in Multimedia Appendix 3. Word pairs having counts larger than 150 were plotted in another network graph. The cutoff of 150 was adopted so that major clusters of word pairs with higher counts could be identified in the network without overly suppressing other pairs with significantly lower counts.

### Latent Dirichlet Allocation Tuning and Model Building

Latent Dirichlet allocation (LDA) [72] is an unsupervised machine learning method that allows observations such as words or documents in a corpus to be explained by latent groups such as topics. LDA has been used in topic modeling of public opinions on certain vaccinations for human papillomavirus (HPV) [73] and influenza virus [74]. However, LDA topic modeling on COVID-19 vaccination was yet to be done. The corpus preprocessed was converted into a document-term matrix, and then terms that were sparse by less than 99.9% were retained for LDA modeling. The R library package *ldatuning* [75] was used to estimate the optimal number of topics in the LDA model. Four different metrics were computed in a range of topics (2-50) to identify the optimal number (Multimedia Appendix 4). The lower the metrics of “Arun2010” [76] and “CaoJuan2009” [77], and the higher the metrics of “Griffiths2004” [78] and “Deveaud2014” [79], indicated a better number of topics to fit the LDA model. In this study, the metric of “Deveaud2014” reached its highest level and the metric of “CaoJuan2009” reached one of the lowest levels at 3 topics that were adopted as the number of topics for LDA modeling. Another R library package *topicmodels* [80] was used to estimate the two posterior Dirichlet distributions—theta distribution over the 3 topics within each tweet and beta distribution over all words within each topic. Only the top 100 words with the highest beta values were visualized using a word cloud for each topic. A larger font size and a higher level of opacity were used to indicate words with higher beta values. In each topic, the top 20 tweets, except those from news sources, with the highest theta values, which were also larger than those of the other two topics for each tweet, were reported.

### Sentiment Analysis

The R library package *syuzhet* [81], which applies Stanford’s CoreNLP [82] on text against an emotion dictionary, was used to score each tweet based on the 2 sentiments and 8 emotions defined in the Canadian National Research Council’s Word-Emotion Association Lexicon [83,84]. There were 10 categories for scoring a tweet. The 2 sentiments were negative and positive, while the 8 emotions were anger, fear, anticipation, trust, surprise, sadness, joy, and disgust. The polarity of a tweet could be positive or negative, whereas emotion recognition aimed to identify the emotions that a tweet carried. If a tweet was associated with a particular emotion or sentiment, it would score points that reflect the degree of valence with respect to that category. Otherwise, it would have no score for that category.

## Results

### Overview

We first analyzed the preprocessed tweets by visualizing the word tokens with a count of >250 in the corpus as shown in the word cloud in Multimedia Appendix 5. The larger the word font size in the cloud, the higher the number of counts in the corpus. The top 10 high-frequency words were “trials,” “australia,” “virus,” “news,” “developers,” “flu,” “people,” “years,” “world,” and “testing.” Following that, other frequently used words included: “research,” “working,” “timeline,” “immune,” “australian,” “effects,” “russian,” “health,” “human,” and “government.” Based on the descriptive statistics of word counts, news about the pandemic, seasonal flu, and vaccine trials were major discussion topics among Australians. Other topics such as the effects of infection control strategies and immunity, the situation overseas, and the government’s responses were also relatively prominent.

**Figure 1:**
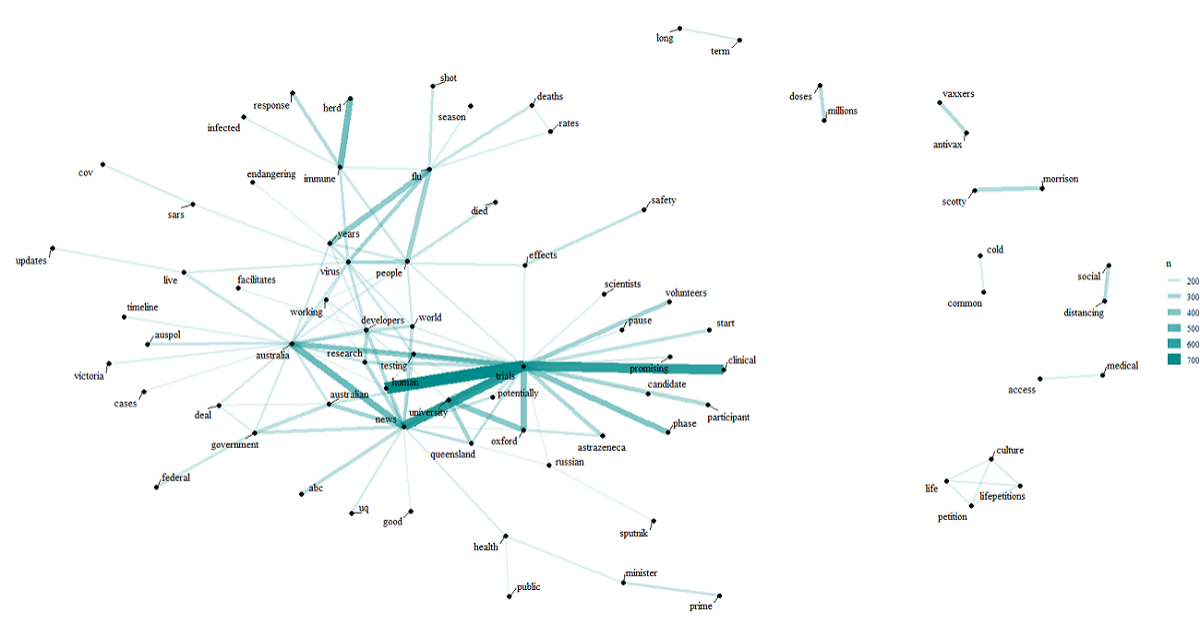
Network of word pairs with counts above 150 in the corpus.

Figure 1 shows the network of word pairs with counts above 150 in the corpus. The word tokens linked with edges, where thicker and more opaque lines indicate a higher number of counts. From the graph, a group of words that were frequently used together were “trials,” “human,” “clinical,” “news,” and “australia.” Moreover, the word “trials” was linked to a number of word tokens such as “phase,” “australia,” “testing,” “volunteers,” and “university”; the latter was linked to “oxford” and “queensland.” Another cluster of words that were commonly used together included “flu,” “years,” “virus,” and “people.” Bigrams such as “herd” and “immune” had some associations with “flu” and “virus.” There were a few word pairs, such as “antivax” and “vaxxers,” which were not connected to the main network and had a relatively small number of counts at the periphery of the graph.

**Figure 2:**
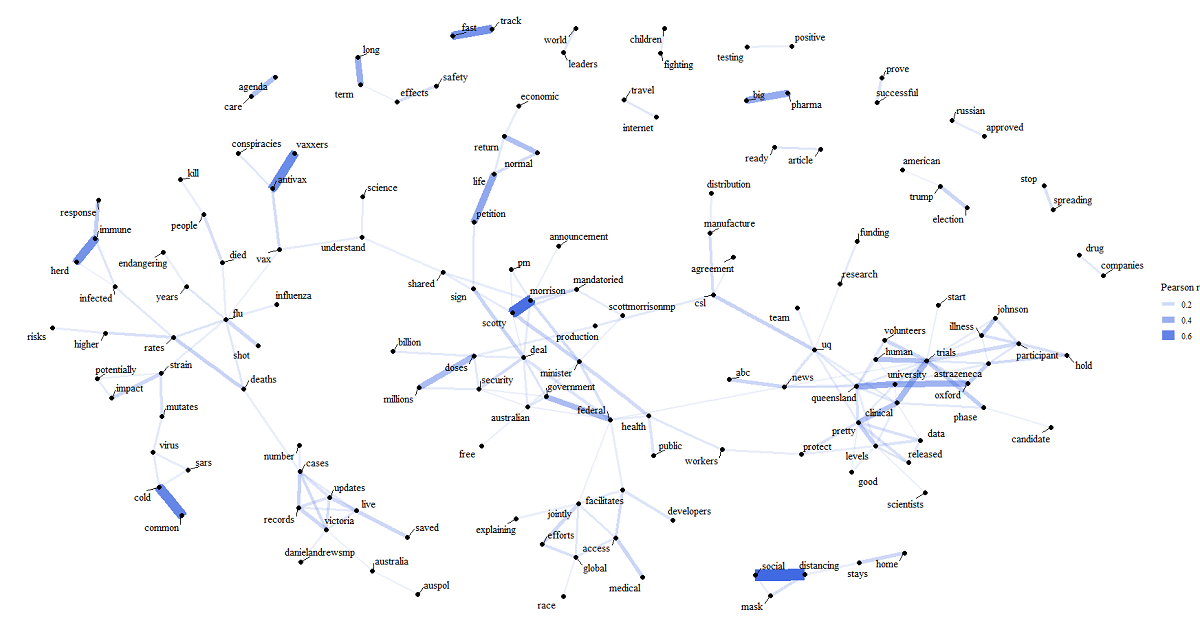
Network of correlations (r>0.10) between word tokens with counts above 250 in the corpus.

We further examined the correlations between word tokens. The network of correlations (r>0.10) between word tokens with a count above 250 in the corpus is visualized in Figure 2, where the edges with a larger width and higher opacity indicate stronger correlations between word tokens. A major network of words consisted of keywords associated with the development and clinical trial of vaccines such as “trials,” “clinical,” “human,” “phase,” “volunteers,” “participant,” “astrazeneca,” “university,” “queensland,” and “oxford.” Another noteworthy major word network was composed of keywords that were related to the Australian government’s partnership with vaccine manufacturers in providing doses for the public: “deal,” “federal,” “government,” “scotty,” “morrison,” “millions,” and “doses.” On the other hand, “flu” was the center of another cluster associated with “influenza,” “deaths,” “rates,” “vax,” and “shot.” Some word pairs like “common” and “cold,” “herd” and “immune,” and “antivax” and “vaxxers” had distal associations with the main network. The pair “antivax” and “vaxxers” had some associations with “conspiracies” and “vax” linking with “flu” and “understand,” which in turn correlated with “science” and “shared.” Furthermore, “social” and “distancing” had a strong correlation, but this bigram, along with a few words that had some associations with them, did not link with the larger network of word tokens. Other similarly independent bigrams included “fast track” and “big pharma.”

We built a 3-topic LDA model and visualized the top 100 probability (beta) distributions of words for each topic in word clouds (Multimedia Appendix 6). The beta values are reported in Multimedia Appendix 7, and the top 20 probability (theta) distributions of topics in the tweet samples are shown in Multimedia Appendices 8-10. Three topic themes were synthesized from the word clouds and tweets extracted.

### Topic 1: Attitudes Toward COVID-19 and COVID-19 Vaccination

The latent topic 1 centered on the public’s attitudes or actions toward COVID-19 vaccination, which were associated with personal values, theories, information received, or personal experiences. Vaccine supporters accepted COVID-19 vaccination because they considered that measures should be taken to cope with the rising number of infections, deaths, health care burden, and costs due to COVID-19. They scorned those who pretended to be experts or posted misinformation such as claiming that deaths from COVID-19 were attributable to other diseases. In addition, they also supported public vaccinations by taking actions such as seeking funding sources and media to promote vaccine trials. Those who worried about the COVID-19 vaccine were skeptical about conspiracy theories such as the “mark of the beast” and microchips in vaccines. The sudden pause of vaccine trials also triggered worries among users about the safety of vaccination. Some Twitter users claimed that they would not get vaccinated because of previous experience with vaccination-related adverse effects. Nonetheless, stock prices increased when positive news about vaccine development were released. Other Twitter users disregarded COVID-19, expressing that COVID-19 had a much lower death rate than the flu, thus making it insignificant for vaccination, which they deemed would only benefit pharmaceutical firms or be politicized. Moreover, implementing lockdowns before mass vaccination was not considered efficient in the long run. Users also thought that COVID-19 should not deserve more attention than other global problems such as climate change, aged care, or other diseases.

### Topic 2: Advocating for Infection Control Measures Against COVID-19

The latent topic 2 indicated that some Twitter users were positive about the development of COVID-19 vaccines and antivirals and recognized the need for these products. Meanwhile, they also advocated following infection control measures and disproved misinformation or conspiracy theories. Some Twitter users rebutted tweets that may have been posted by antivaxxers or conspiracy theorists. For example, these users refuted skepticism over the safety of the rapidly produced vaccines, false claims about the association between the flu vaccine and COVID-19 infections and deaths, and inaccurate beliefs about vaccination coverage for achieving herd immunity differing across diseases. Some of their tweets emphasized the rising number of deaths related to COVID-19 within a rather short period compared with other pandemics in the past. They argued that although there were deaths caused by the flu, there were drugs, vaccines, and promotion campaigns targeting the flu. In comparison, deaths from COVID-19 were soaring, and even worse than the flu, without mass vaccinations or antivirals. However, COVID-19 deaths could have been preventable. With previous experiences in developing vaccines for other coronaviruses such as MERS-CoV, users believed that the COVID-19 vaccine could be successfully developed to protect vulnerable groups like patients. They believed that everyone was susceptible to COVID-19 after contracting the coronavirus without vaccination. In the future, antivirals could also be developed. Beyond vaccines and drugs, they thought physical measures such as wearing masks and social distancing should be followed, particularly at a time when mass vaccination and antivirals are not yet available.

### Topic 3: Misconceptions and Complaints About COVID-19 Control

The latent topic 3 generally showed the baseless claims and conspiracy theories that antivaxxers held against the COVID-19 vaccine as well as complaints and helplessness about testing and lockdown measures, which would likely end with vaccination-induced herd immunity. Some Twitter users made claims that were unfounded or based on conspiracy theories against the COVID-19 vaccine. For example, one concluded that Australia suggested using a vaccine that had never been tested or certified to fight the virus. Some others believed that hydroxychloroquine was an effective treatment; hence, banning its use was viewed as a politicized action. Users also thought that those rejecting hydroxychloroquine should take vaccines from Bill Gates, who was falsely accused of planning to implant microchips into human bodies via vaccinations. However, other Twitter users pointed out the limitations of vaccinations such as their inability to prevent viral transmissions or treat COVID-19 and its complications. Even if vaccines are available, a high number of doses globally and tests for the virus or even antibodies are required if COVID-19 is not eradicated. Some complained that the tests led to an increase in known positive cases and in turn a prolonged lockdown, making the situation helpless without the availability of a vaccine. On the other hand, provaxxers celebrated the success in vaccine development. They criticized antivaxxers for not believing in science and accepting vaccination, as well as for disregarding the serious consequences of COVID-19 and for suggesting natural herd immunity, which would be catastrophic. For example, allowing the rampant spread of the coronavirus would lead to health care system breakdown and loss of life.

**Figure 3:**
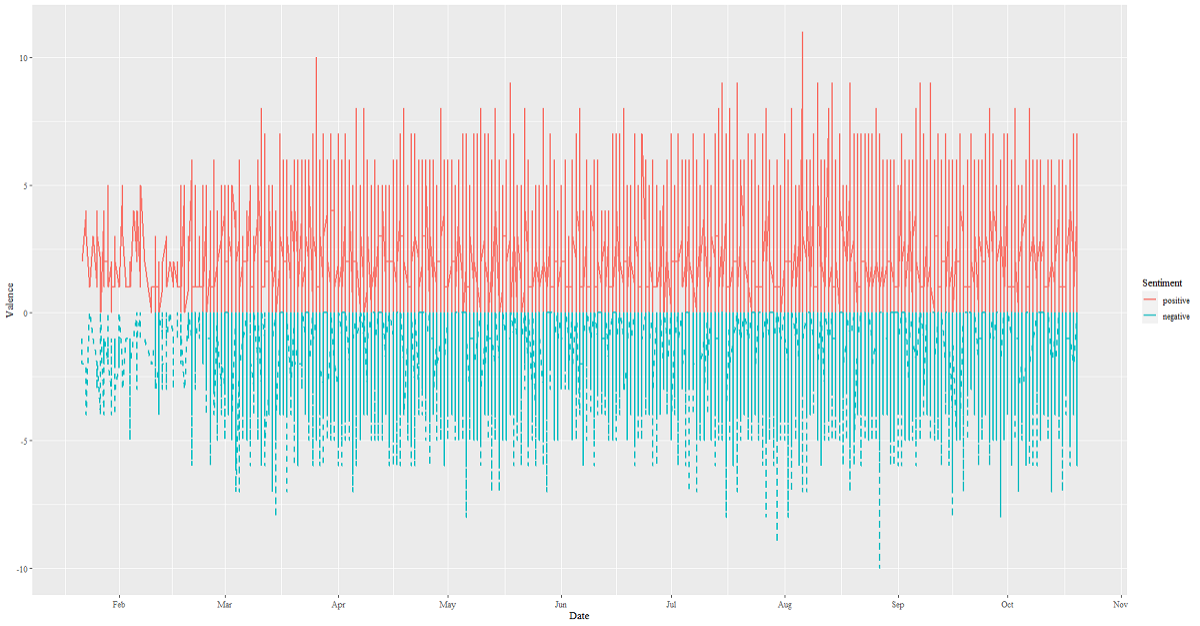
Distributions of sentiment valences between January and October 2020.

Figure 3 shows the change in sentiment scores of all tweets between January and October 2020. In each tweet, there could be both positive and negative sentiment with valences in opposite directions. Figure 3 shows that the scores increased gradually between January and March 2020. The higher the sentiment score, regardless of direction, the likelier the tweet will have stronger sentiments. However, most tweets expressed positive sentiment (score=62,498, 67%) rather than a negative one (score=27,622, 30%), while 940 (3%) tweets were neutral.

**Figure 4:**
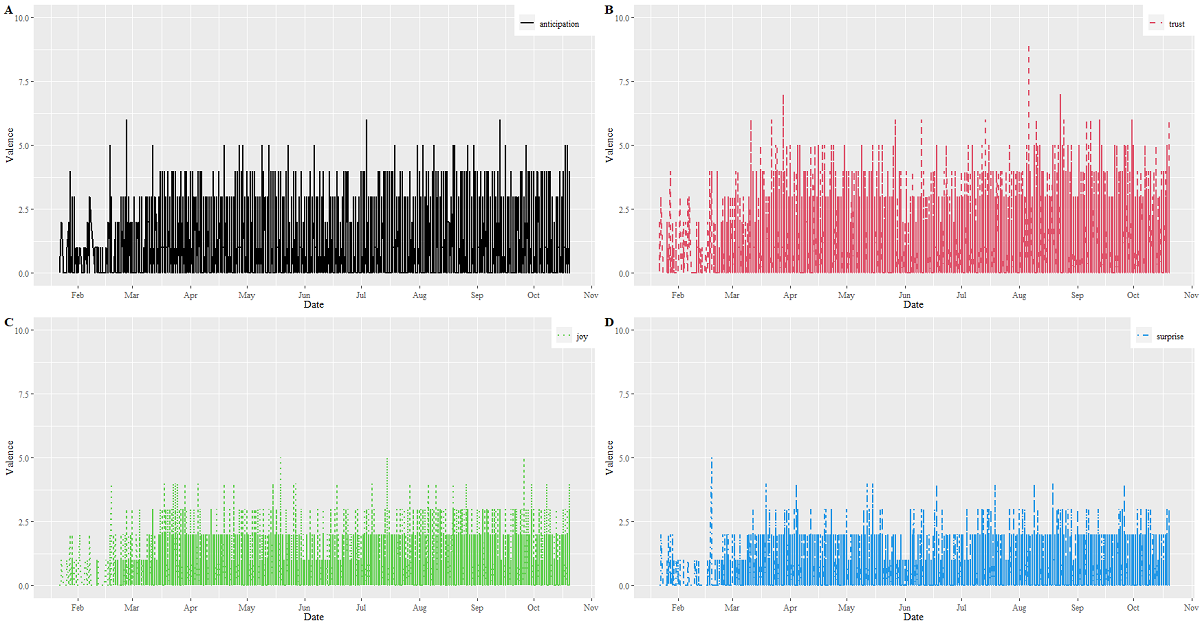
Distributions of emotion valences for (A) anticipation, (B) trust, (C) joy, and (D) surprise between January and October 2020.

Figure 4 shows the emotion scores with respect to anticipation, joy, surprise, and trust in all tweets. The scores also rose in the first quarter of 2020. Approximately 45% of the scores were associated with these 4 emotions. Specifically, the emotion components were trust (score=22,436, 17%) and anticipation (score=19,278, 14%). Some tweets scored for surprise (score=7865, 6%) and joy (score=10,296, 8%).

**Figure 5:**
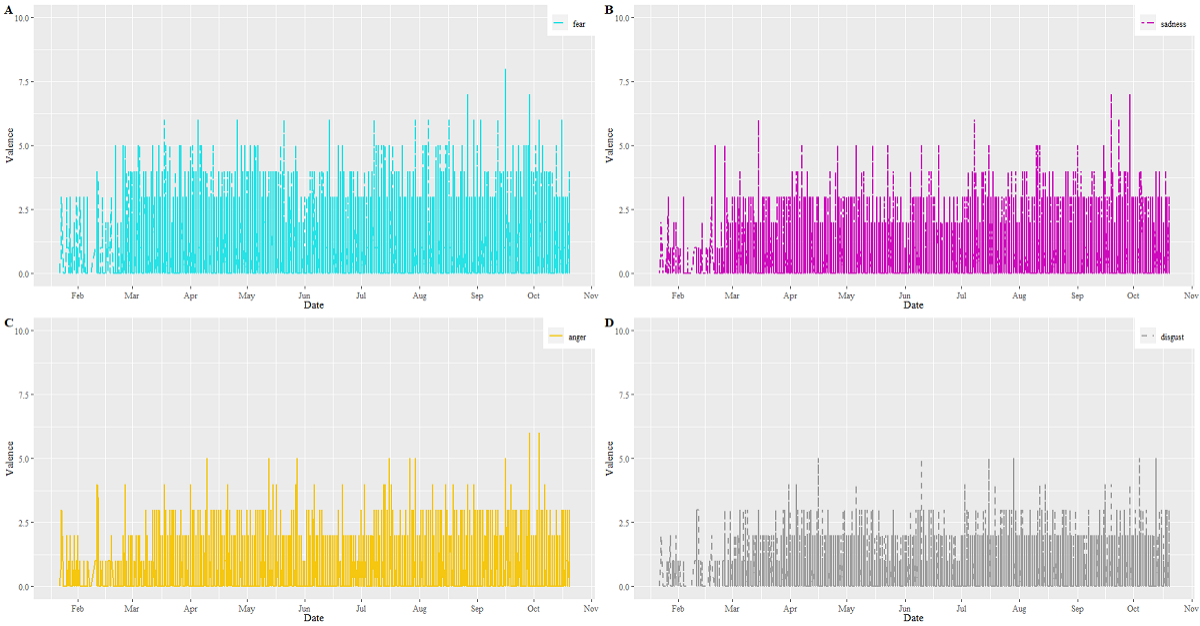
Distributions of emotion valences for (A) fear, (B) sadness, (C) anger, and (D) disgust between January and October 2020.

Figure 5 shows the scores of negative emotions such as anger, disgust, fear, and sadness for all tweets. The scores increased in the first 3 months of 2020; approximately one-third of the scores were associated with these negative emotions. Among them, fear was the most significant one (score=18,449, 14%). Other emotions included sadness (score=11,082, 8%), anger (score=9091, 7%), and disgust (score=6337, 5%). On the other hand, nearly 22% (n=6994) of the tweets were emotionally neutral.

## Discussion

### Principal Findings

We found that the public opinion about COVID-19 vaccines fell under 3 latent topics among Australian Twitter users from January 22 to October 20, 2020. Topic 1 was about different attitudes and actions toward COVID-19 and its vaccination. Provaxxers recognized the consequences of the COVID-19 pandemic and supported vaccine trials. Those who were skeptical about vaccines were affected by misinformation and adverse effects, which are statistically rare. Some Twitter users gave low priority to COVID-19 and hence vaccination against it and other unrelated problems. Topic 2 showed that some Twitter users advocated for infection control measures, had confidence in COVID-19 vaccine trials, and rebutted tweets that were derived from conspiracy theories or misinformation. They argued that infections and deaths from COVID-19 had overtaken previous pandemics, and other measures such as wearing masks and social distancing should be followed when mass vaccination is yet to come. Topic 3 centered on baseless claims, conspiracy theories, complaints, and misconceptions about various measures against COVID-19, including vaccines, drugs, virus testing, lockdown, and herd immunity. The major pitfall of these tweets was that their content could not be supported with any valid scientific evidence; further, the complaints were not directly associated with any solutions. Another significant finding was that nearly two-thirds of the sentiments in the tweets related to COVID-19 vaccines were positive. Of those tweets analyzed, 17% of the emotions were linked with trust and 14% were associated with anticipation. However, 14% contained fear and 8% expressed sadness. Overall, less than one-third of the tweets’ sentiments were classified as negative, and one-third of the tweets were associated with the 4 negative emotions (ie, fear, sadness, anger, and disgust).

### Comparison With Prior Work

In the past decade, machine learning has been applied to explore topics and sentiments of content from Twitter users about vaccinations. Some studies have examined tweets related to vaccinations in general, while others have analyzed vaccination-related tweets focusing on a particular virus or disease, such as the influenza virus, which causes respiratory illness, or HPV, which is mainly sexually transmitted. Those studies identified both positive and negative sentiments toward vaccinations, as well as neutral sentiment. Nevertheless, the outcomes of sentiment categories and the topics identified from Twitter users varied across studies focusing on different countries, years, viruses, and thus diseases.

For example, Jamison et al [85] generated 100 topics using LDA in which nearly half were annotated as provaccination, and less than 30% were coded as antivaccination from English, vaccine-relevant tweets between 2014 and 2017. However, Raghupathi et al [60] found that both positive and negative sentiments accounted for 40% of English tweets in the first half of 2019. On the other hand, the composition of sentiments in non-English tweets could be different from of English tweets. In Italy, Tavoschi et al [51] used support vector machine to classify tweets’ term frequency–inverse document frequency between 2016 and 2017, and found that 60% were neutral, 23% were against vaccination, and only 17% were provaccination. It was also found that the number of provaccine tweets became greater than the number of antivaccine tweets when news about compulsory vaccination and the soaring rate of positive cases or deaths were broadcast [86].

The topics identified were not entirely similar across studies. For instance, Jamison et al [85] summarized 5 provaccine themes and 5 antivaccine themes from 100 topics; and Raghupathi et al [60] identified 3 focus areas (eg, the search for better vaccines, the disease outbreak, and debates between provaxxers and antivaxxers regarding measles). Chan et al [74], who studied influenza vaccination in the United States, used LDA to create 10 topics in which some shared similar attributes with the themes of Jamison et al [85], such as vaccine science, safety concerns, and conspiracy theories. Some, but not all, of the similar themes, focus areas, and topics could also be seen in the analyses of tweets about vaccination regardless of virus types such as those in the studies surrounding HPV vaccinations [73,87-90].

### Added Value of This Study

This study is the first topic modeling and sentiment analysis of tweets in Australia about COVID-19 vaccinations. As COVID-19 has turned into a pandemic, it is necessary to explore and summarize public opinion and sentiments pertaining to discussions on the COVID-19 vaccine, so as to prepare for the promotion of vaccination, which needs to be strengthened. This study used a traditional natural language processing technique—LDA—to identify 3 latent topics in the tweets associated with COVID-19 vaccinations: (1) attitudes toward COVID-19 and its vaccination, (2) advocating infection control measures against COVID-19, and (3) misconceptions and complaints about COVID-19 control. Furthermore, this study discovered that positive sentiment in COVID-19 vaccine discussions was higher than negative sentiment, and trust and anticipation comprised relatively large proportions of the emotions observed, as well as fear. This study visualized results using word clouds, counts of word pairs, and correlations between words, which offer supplementary angles in interpreting the results. For example, high-frequency words and word pairs that commonly appeared together were intuitively presented.

The Australian population has been the focus of research on tweets related to vaccination in previous studies. Taking the HPV vaccine as an example, nearly one-fifth of Australian Twitter users expressed health concerns about the vaccine [88], and around one-third of the exposure to information on Twitter was associated with misinformation or adverse effects of the vaccine [89]. Our study provides new insights into topics of discussion in Australia and sentiments toward vaccination against COVID-19, which is now a global pandemic and has caused over 900 deaths in Australia [3] and over 1.8 million deaths worldwide [4] as of early January 2021. By assessing public opinion and the sentiments associated with COVID-19 vaccination, governments and health agencies can plan, tailor, and implement a timely promotion of vaccination to achieve herd immunity as soon as possible.

### Implications

In the results of the previous studies, we did not see a prevalent objection or opposition, in terms of topics identified or sentiments, toward vaccination regardless of virus types. A number of topics’ focus areas or themes shared a certain level of similarity across studies concerning different viruses. For instance, topics of safety, scientific evidence, and conspiracy theories were commonly found across studies. Topics like scandals associated with vaccines, misinformation, and disease outbreaks were identified in some other studies. These results indicated public concern about the benefits and risks of vaccination at the individual and social levels, and the type of virus or disease when deciding whether to receive a vaccine or not.

In our study, besides fabricated information such as microchips in vaccines and the flu vaccine causing COVID-19 deaths, some Twitter users thought that COVID-19 was not serious enough compared to other existing global crises, and that the pandemic was being politicized or commercialized. These conspiracy theories, along with other antivaccine propagandas such as encouraging natural herd immunity, indicated that the risks of deaths, complications, or sequela arising from COVID-19 to others, or to oneself, were acceptable to some members of the public.

Although the Australian opinion showed more positive sentiment related to COVID-19 vaccinations, the positive sentiment was not a leading majority compared to the negative one. This means more work needs to be done to promote vaccination so as to achieve herd immunity to protect vulnerable and minority groups. Rigorous science that is easily understandable needs to replace biased, fabricated, or outdated information in the public. Governments should build and strengthen the public’s confidence in COVID-19 vaccination, if it is not mandatory, that is, required by law, beyond arranging vaccine delivery logistically and vaccine administration clinically.

### Limitations

Our results represent Twitter users in the Australian public, which is a different approach from national survey statistics. However, the public opinions collected on Twitter may represent views from younger populations. Previous studies showed that around 85% to 90% of Twitter users were aged less than 25-40 years, which varied across locations such as the Netherlands [91], the United Kingdom [92] and other places [93]. Older adults’ opinions require further investigations with modifications to the study design whereas younger adults’ opinions on the vaccine deserve continuous attention. Goldstein et al [94] reported that those aged less than 35 years had high cumulative rates of COVID-19 infections in the community where transmissions in secondary schools or high schools were robust. A report published by the US Centers for Disease Control and Prevention [95] showed that the percent positivity of SARS-CoV-2 RT-PCR (reverse transcription–polymerase chain reaction) tests increased early among young people, followed by a rise in positivity in middle-aged and older adults. Consequently, around 20% of adolescents manifested symptoms compared with nearly 70% of the elderly [96], who are subject to a higher probability of further developments leading to death. Hence, there is an urgent need to explore younger population’s opinion and acceptability of vaccination, which could have significant impact on disease control in the first place.

In addition to the study period and the country of concern, analysis methods might lead to variation in topics and sentiments toward vaccinations. For supervised learning such as support vector machine, a training set is required, which needs to be manually labeled; this might carry some subjectivity in categorizing tweets into predefined topics for training. However, the advantage is that the set could be used to validate the model performance and then test a large data set. Considering unsupervised learning such as LDA, Dirichlet multinomial mixtures (DMM), and k-means of term frequency–inverse document frequency, the primary limitation is the subjectivity in defining the topics created [60,74]. In addition, a sound reason or calculation is needed to support the preset number of topics, which would affect the results.

Some previous studies generated a rather high number of topics (30-100) using an LDA or DMM model, and then manually grouped the topics into themes [73,85,89]. However, there was risk of bias since the content of each topic was not reported in detail, and the contents of the themes could be mixed, which is difficult to interpret. Furthermore, the manual grouping also contained the risk of subjectivity. In the current study, we adopted LDA, which was similar to the one used by Chan et al [74]. We identified 3 latent topics in which the importance of words were visualized; the frequency of word pairs and correlations between words provided additional results corresponding to the topic content.

Regarding sentiment analysis, the number of emotion categories were limited to 8 [83,97], but emotion is an abstract and broad concept that may involve as many as 27 categories [98]. Furthermore, words with spelling mistakes could not be identified and analyzed in the algorithm. With respect to each term for the development of an emotion lexicon by Mohammad and Turney [83], only 5 individuals in the public were recruited to annotate a term against each of the 8 emotions. The emotions of a term were annotated without considering possible contexts. Moreover, the interrater reliability statistics were not reported though the agreement percentages were apparently high.

### Future Directions

Our study adopted an unsupervised machine learning method—LDA—for topic analysis. Future studies could investigate supervised learning to train classifiers to categorize tweets into different topics and sentiments based on a recognized theoretical framework. Such a framework could be proposed after an extensive literature review and qualitative synthesis; manual annotations should be as transparent, objective, and reliable as possible. Results from supervised learning following the same theoretical framework could be compared across the analyses of different data sets, for example, the results from different countries as shown by Shapiro et al [88]. Public opinions across countries require further study. For instance, recent online surveys of US adults found that only half claimed that they were “very likely” to get the COVID-19 vaccine [99], and one-third would not accept recommendations for vaccination [100]. In the United Kingdom, around one-third of the adult sample showed hesitancy or resistance against COVID-19 vaccination [101,102]. In the future, a spatiotemporal analysis of tweets about COVID-19 vaccination could be attempted. Similar studies have been conducted on Twitter data to study emergency department visits for influenza-like illness in New York City [103], COVID-19–related stress symptoms in the United States [104], and communicating the risk of MERS infections in South Korea [105]. Furthermore, individual reactions toward the COVID-19 vaccine in tweets could be monitored over time and tested for correlations between frequencies of identified topics or emotions, important real events, and health indicators such as vaccination coverage, infection rate, and death rate. In addition to studying the spread of misinformation and conspiracy theories on social media, future research should explore personal values that might hinder collective health care strategies and positive outcomes.

### Conclusions

Our findings indicate that the Australian public possessed varying attitudes toward COVID-19 and its vaccination. Moreover, some had misconceptions and complaints about COVID-19 and infection control measures, while others advocated for pharmaceutical and nonpharmacological measures against COVID-19. Nonetheless, in our sentiment analysis, the level of positive sentiment in public opinion may not be strong enough to further a high vaccination coverage to achieve vaccination-induced herd immunity, which is essential to protect oneself and others. For those without contraindications, getting vaccinated is not merely a personal choice but is also a way of protecting the community. Governments should explore public opinion and sentiments toward COVID-19 vaccination and get the public psychologically prepared for vaccination with evidence-based, authorized, and understandable information, in addition to supporting the biomedical development, storage, delivery, and clinical administration of vaccines.

## Appendix

Multimedia Appendix 1 Number of tweets collected between January 22 and October 20, 2020.

Multimedia Appendix 2 Plot of word tokens against counts sorted.

Multimedia Appendix 3 Plot of word pairs against counts sorted.

Multimedia Appendix 4 Plot of number of topics against LDA tuning scores.

Multimedia Appendix 5 Word tokens with counts above 250 in the corpus.

Multimedia Appendix 6 Word distributions over 3 topics in the latent Dirichlet allocation model.

Multimedia Appendix 7 Top 100 probability (beta) distributions of words in each topic.

Multimedia Appendix 8 Top 20 probability (theta) distributions of topic 1 in tweet samples.

Multimedia Appendix 9 Top 20 probability (theta) distributions of topic 2 in tweet samples.

Multimedia Appendix 10 Top 20 probability (theta) distributions of topic 3 in tweet samples.

## Abbreviations

API: application programming interface
DMM: Dirichlet multinomial mixtures
HPV: human papillomavirus
LDA: latent Dirichlet allocation
MERS: Middle East respiratory syndrome
MERS-CoV: Middle East respiratory syndrome coronavirus
MMR: measles, mumps, and rubella
NASA: National Aeronautics and Space Administration
R_0_: reproductive number
NLP: natural language processing
PCV13: 13-valent pneumococcal conjugate vaccines
RT-PCR: reverse transcription–polymerase chain reaction
SAO: Smithsonian Astrophysical Observatory
SARS: severe acute respiratory syndrome
SARS-CoV: severe acute respiratory syndrome coronavirus
