# Supplementary figures and images for: Tweet Topics and Sentiments Relating to COVID-19 Vaccination Among Australian Twitter Users: Machine Learning Analysis
Source: J Med Internet Res. 2021 May 19;23(5):e26953. doi: 10.2196/26953 (PMC8136408; doi:10.2196/26953)

**Multimedia Appendix 1**. Number of tweets collected between January 22 and October 20, 2020.


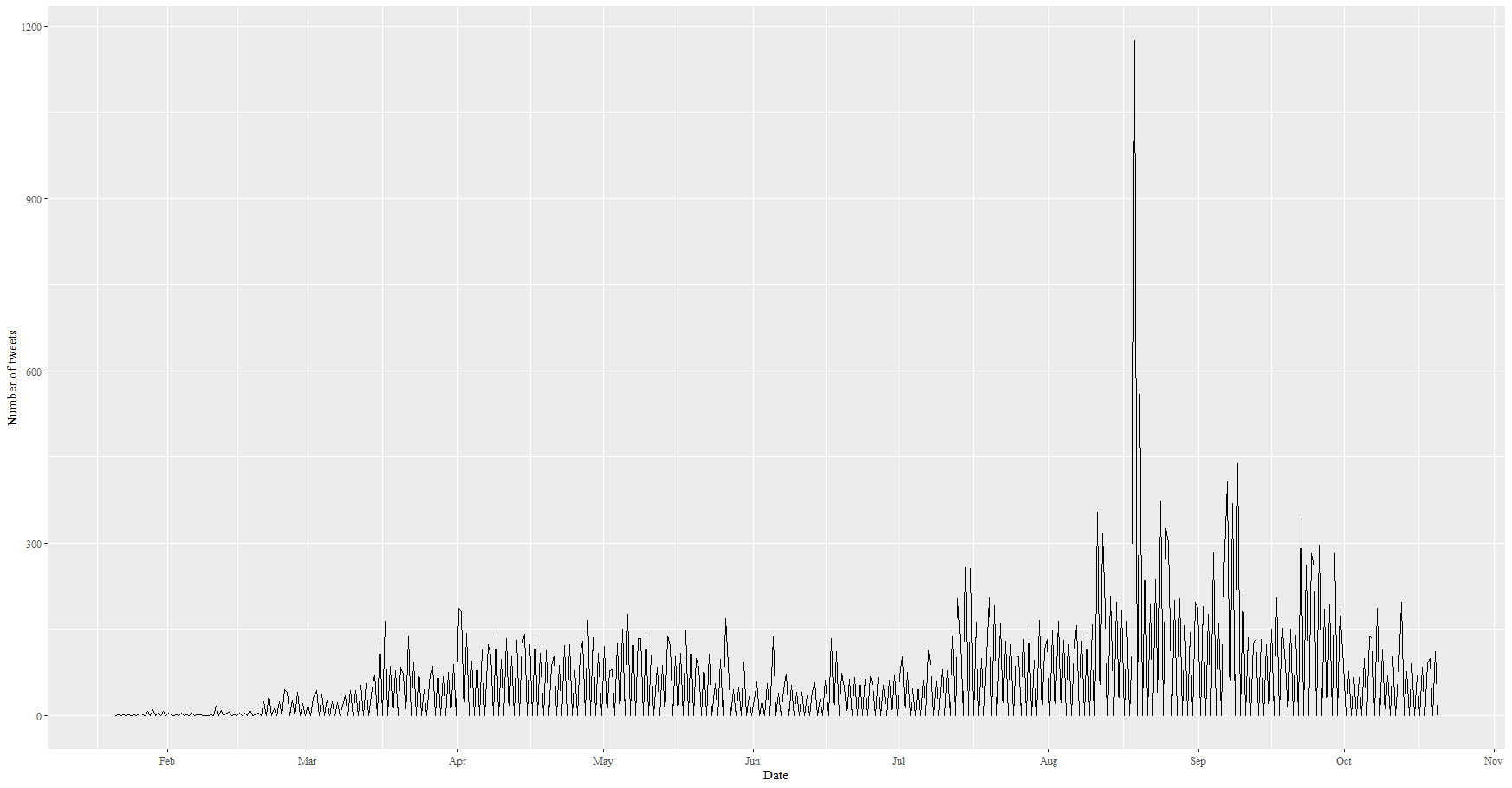

Supplement: Multimedia Appendix 1 [file jmir_v23i5e26953_app1.doc]

**Multimedia Appendix 1.** Plot of word tokens against counts sorted.


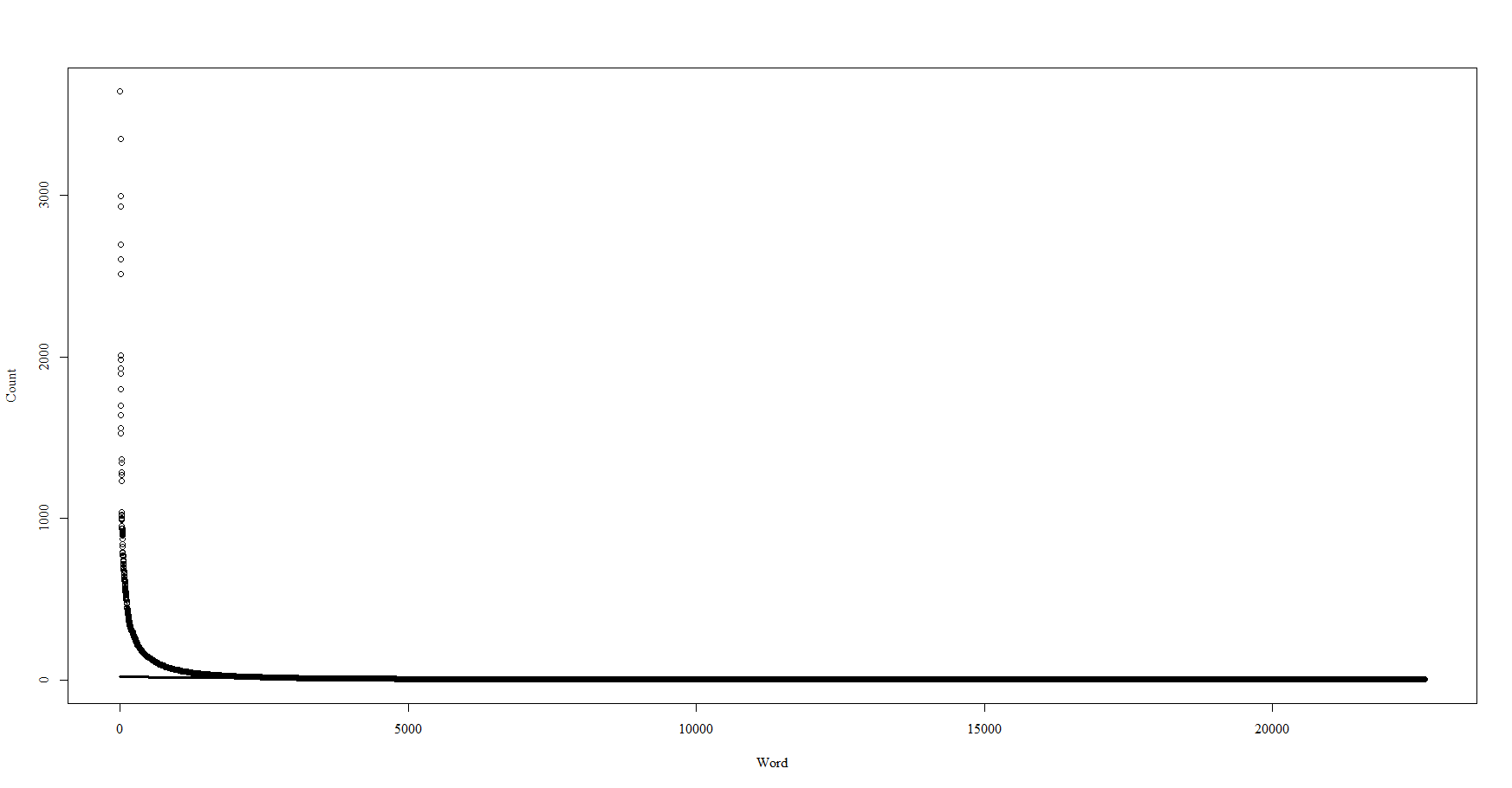

Supplement: Multimedia Appendix 2 [file jmir_v23i5e26953_app2.doc]

**Multimedia Appendix 1.** Plot of word pairs against counts sorted.


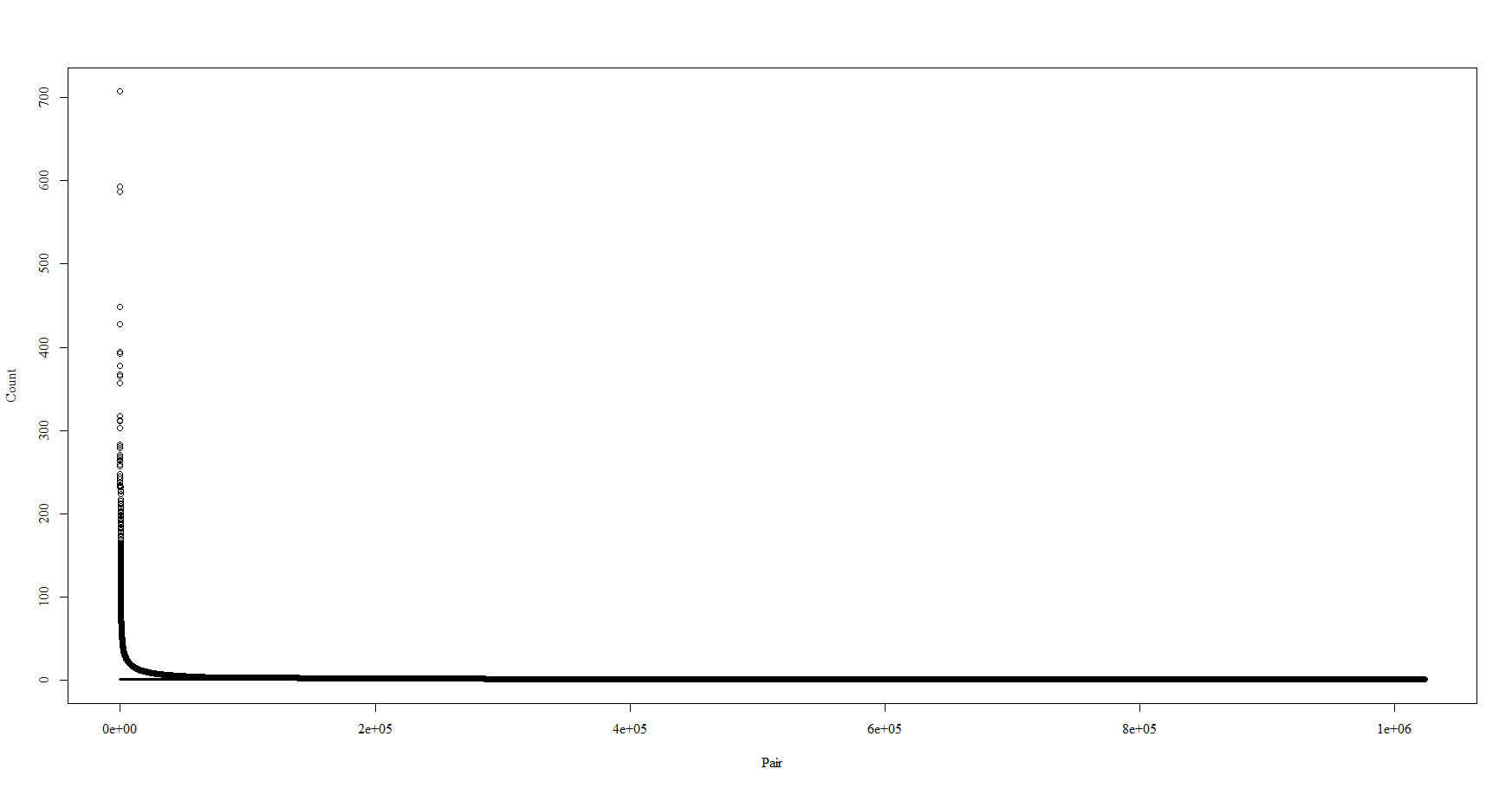

Supplement: Multimedia Appendix 3 [file jmir_v23i5e26953_app3.doc]

**Multimedia Appendix 1.** Plot of number of topics against LDA tuning scores.


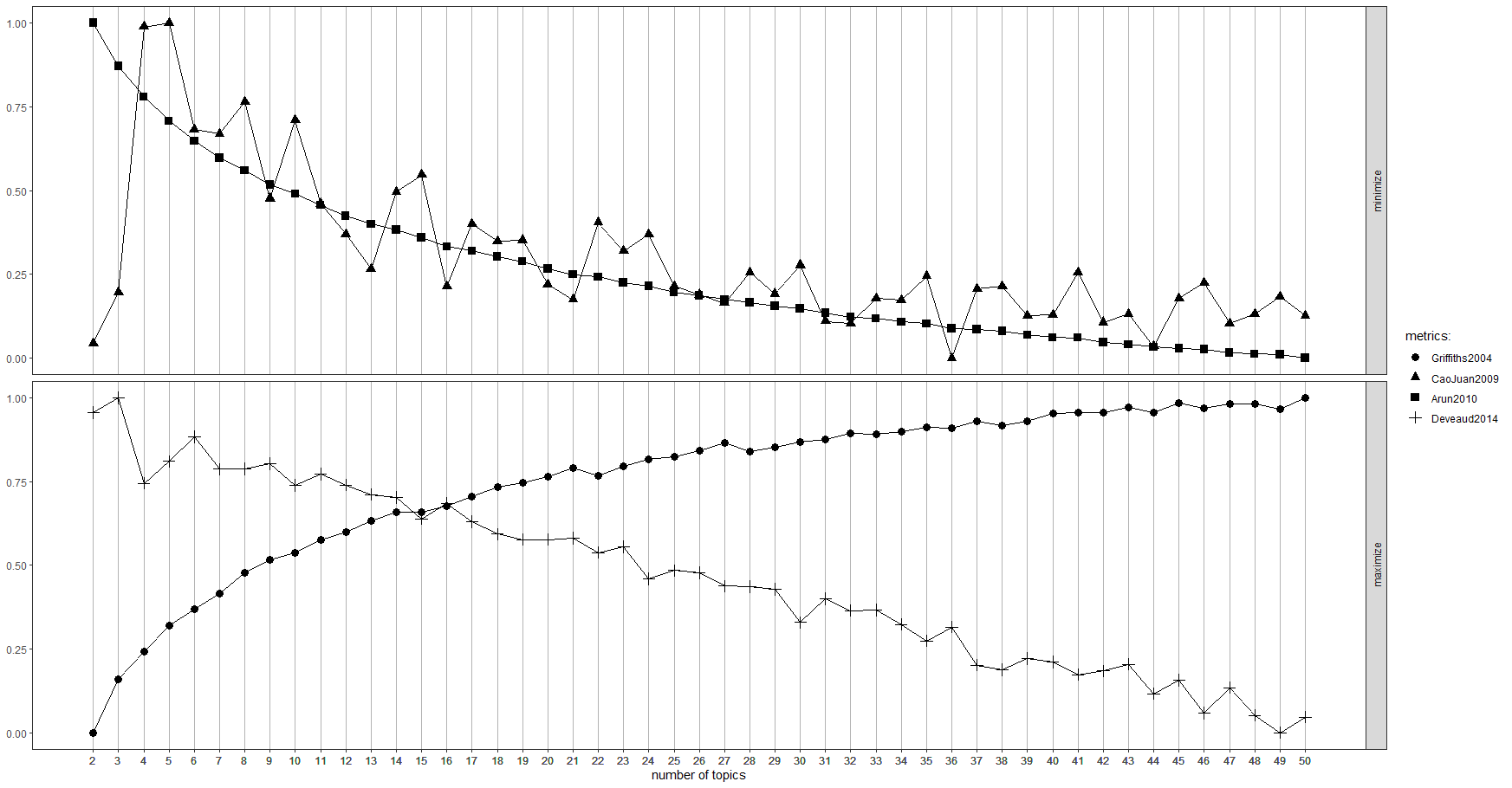

Supplement: Multimedia Appendix 4 [file jmir_v23i5e26953_app4.doc]

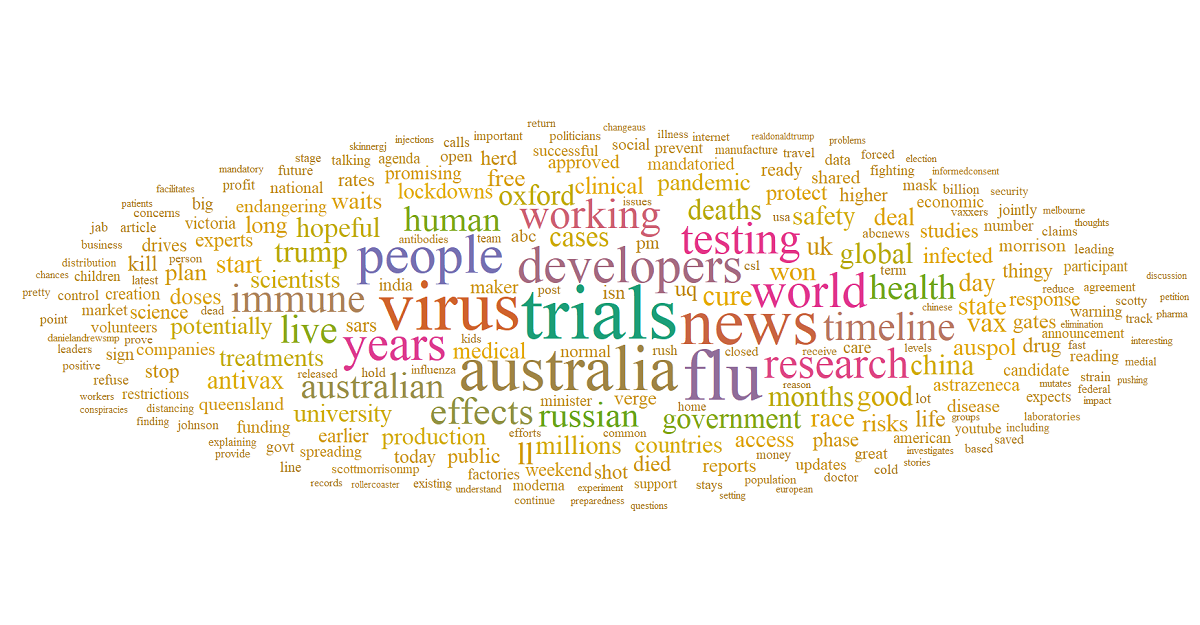

Supplement: Multimedia Appendix 5 [file jmir_v23i5e26953_app5.png]

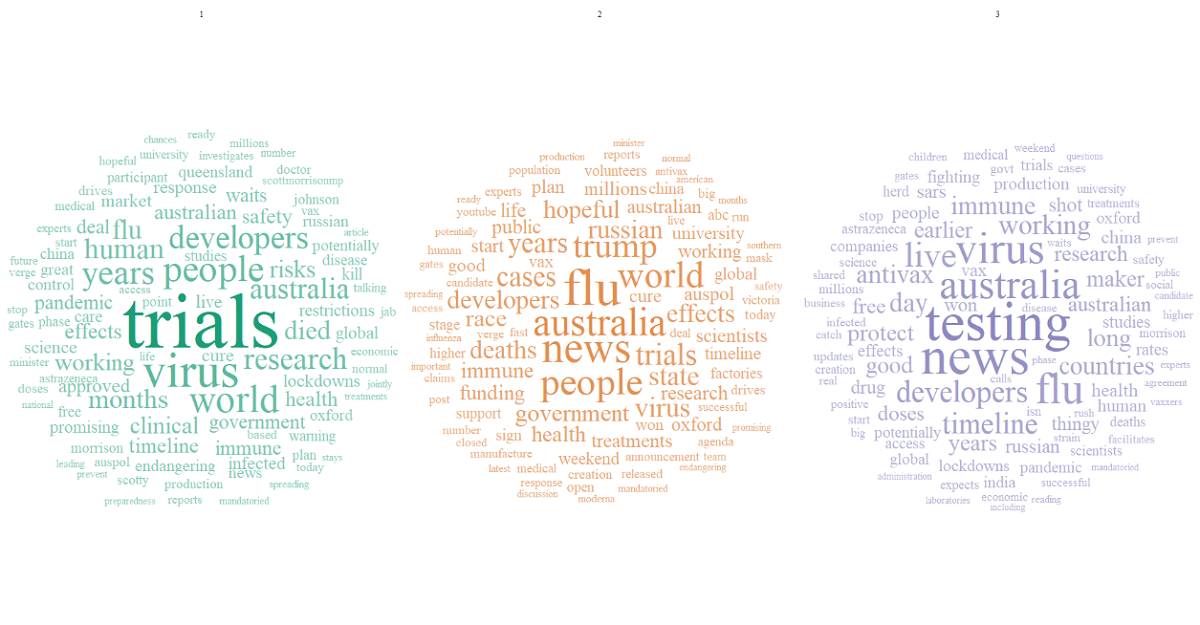

Supplement: Multimedia Appendix 6 [file jmir_v23i5e26953_app6.png]
