# Supplementary material for: Tweet Topics and Sentiments Relating to COVID-19 Vaccination Among Australian Twitter Users: Machine Learning Analysis
Source: J Med Internet Res. 2021 May 19;23(5):e26953. doi: 10.2196/26953 (PMC8136408; doi:10.2196/26953)
Supplement: Multimedia Appendix 7 [file jmir_v23i5e26953_app7.docx]

**Multimedia Appendix 7.** Top 100 probability (beta) distributions of words in each topic.

| Topic 1 |  | Topic 2 |  | Topic 3 |  |
| --- | --- | --- | --- | --- | --- |
| term | beta | term | beta | term | beta |
| trials | 0.029605405 | australia | 0.013995 | testing | 0.020541 |
| virus | 0.014339705 | flu | 0.013172 | news | 0.017094 |
| people | 0.011910221 | news | 0.012781 | australia | 0.014711 |
| world | 0.011494524 | people | 0.012264 | virus | 0.012843 |
| developers | 0.011396044 | world | 0.010691 | developers | 0.010746 |
| research | 0.009819939 | trump | 0.009855 | flu | 0.010729 |
| years | 0.009760756 | developers | 0.008363 | timeline | 0.009147 |
| australia | 0.009225427 | trials | 0.008194 | live | 0.008497 |
| human | 0.008020217 | government | 0.008 | working | 0.008396 |
| months | 0.007824672 | cases | 0.007564 | countries | 0.007653 |
| working | 0.006979976 | years | 0.007391 | antivax | 0.007346 |
| died | 0.00649738 | effects | 0.007307 | immune | 0.00731 |
| clinical | 0.006210546 | deaths | 0.007265 | day | 0.006311 |
| government | 0.006054426 | state | 0.007097 | australian | 0.006299 |
| australian | 0.005948586 | russian | 0.007014 | protect | 0.006289 |
| risks | 0.005899491 | hopeful | 0.006987 | research | 0.006255 |
| effects | 0.00583242 | virus | 0.006741 | long | 0.006007 |
| pandemic | 0.005755502 | immune | 0.005897 | good | 0.005813 |
| timeline | 0.005587399 | health | 0.005688 | earlier | 0.005798 |
| flu | 0.005584583 | treatments | 0.005647 | maker | 0.005725 |
| approved | 0.00539052 | funding | 0.005645 | years | 0.0056 |
| immune | 0.005333685 | race | 0.005522 | doses | 0.005365 |
| safety | 0.005327766 | scientists | 0.00549 | russian | 0.004728 |
| health | 0.005218148 | australian | 0.005376 | thingy | 0.004712 |
| lockdowns | 0.004807243 | research | 0.005364 | free | 0.004531 |
| waits | 0.004781915 | working | 0.005021 | production | 0.00446 |
| response | 0.004677666 | public | 0.004846 | lockdowns | 0.004367 |
| restrictions | 0.004615621 | university | 0.004831 | health | 0.004296 |
| endangering | 0.00457321 | millions | 0.004817 | people | 0.004293 |
| queensland | 0.00451522 | auspol | 0.004491 | shot | 0.004266 |
| market | 0.004463092 | timeline | 0.004475 | fighting | 0.004253 |
| deal | 0.00441127 | weekend | 0.00442 | potentially | 0.004218 |
| promising | 0.004403165 | oxford | 0.004361 | effects | 0.004111 |
| infected | 0.004363318 | start | 0.004196 | human | 0.004094 |
| global | 0.004186094 | global | 0.004157 | studies | 0.004067 |
| science | 0.004151156 | good | 0.004061 | companies | 0.004006 |
| potentially | 0.00412156 | plan | 0.003984 | drug | 0.003981 |
| russian | 0.003707832 | life | 0.003907 | pandemic | 0.003965 |
| participant | 0.003672828 | cure | 0.003851 | china | 0.003951 |
| disease | 0.0036518 | volunteers | 0.003782 | won | 0.00392 |
| control | 0.003629805 | factories | 0.003648 | sars | 0.003881 |
| warning | 0.003532918 | support | 0.003489 | astrazeneca | 0.003841 |
| cure | 0.003511333 | announcement | 0.003435 | access | 0.003545 |
| great | 0.003496202 | china | 0.003304 | oxford | 0.003512 |
| oxford | 0.00342378 | sign | 0.003224 | scientists | 0.0035 |
| studies | 0.003416186 | stage | 0.003147 | rates | 0.003459 |
| care | 0.003393992 | higher | 0.003128 | trials | 0.003436 |
| live | 0.003387638 | creation | 0.003006 | global | 0.003406 |
| production | 0.003339507 | vax | 0.003003 | india | 0.003381 |
| news | 0.003313603 | won | 0.002974 | vax | 0.003301 |
| johnson | 0.003296491 | manufacture | 0.002962 | medical | 0.003292 |
| astrazeneca | 0.003244863 | released | 0.002936 | treatments | 0.003204 |
| morrison | 0.003227929 | candidate | 0.002905 | millions | 0.003051 |
| china | 0.003136001 | medical | 0.00288 | deaths | 0.003042 |
| phase | 0.003038486 | today | 0.002866 | updates | 0.002942 |
| scottmorrisonmp | 0.003020877 | agenda | 0.002821 | university | 0.002927 |
| kill | 0.002922035 | population | 0.002808 | morrison | 0.002917 |
| investigates | 0.002875264 | victoria | 0.002768 | herd | 0.002813 |
| university | 0.002859464 | abc | 0.002735 | safety | 0.002811 |
| point | 0.002826972 | successful | 0.002725 | science | 0.002806 |
| free | 0.002820787 | open | 0.002725 | expects | 0.002805 |
| auspol | 0.002781993 | experts | 0.002688 | creation | 0.002775 |
| drives | 0.002760809 | drives | 0.002678 | stop | 0.002687 |
| economic | 0.002726885 | youtube | 0.002656 | infected | 0.002683 |
| medical | 0.002725319 | reports | 0.002646 | successful | 0.002681 |
| talking | 0.002682161 | production | 0.002513 | business | 0.002658 |
| scotty | 0.002667427 | potentially | 0.002459 | positive | 0.002629 |
| plan | 0.002640828 | human | 0.002443 | facilitates | 0.002615 |
| experts | 0.002620385 | important | 0.002438 | disease | 0.0026 |
| based | 0.002608058 | number | 0.002433 | govt | 0.002559 |
| hopeful | 0.002602846 | response | 0.002428 | economic | 0.002555 |
| minister | 0.002587874 | mandatoried | 0.002404 | cases | 0.002529 |
| doctor | 0.002568622 | big | 0.002355 | weekend | 0.002515 |
| normal | 0.002556661 | antivax | 0.002344 | shared | 0.002448 |
| reports | 0.002547932 | verge | 0.002336 | social | 0.002429 |
| gates | 0.002529514 | claims | 0.002333 | higher | 0.002402 |
| doses | 0.002499905 | access | 0.002333 | children | 0.002382 |
| mandatoried | 0.002473263 | moderna | 0.0023 | start | 0.00236 |
| access | 0.002463569 | spreading | 0.002274 | strain | 0.002342 |
| spreading | 0.002462598 | gates | 0.002273 | agreement | 0.002248 |
| treatments | 0.002354843 | deal | 0.002272 | catch | 0.002234 |
| millions | 0.00235314 | influenza | 0.002264 | gates | 0.002227 |
| start | 0.002328846 | endangering | 0.002258 | calls | 0.0022 |
| today | 0.0023287 | safety | 0.002241 | administration | 0.002199 |
| ready | 0.00231141 | mask | 0.002235 | waits | 0.002187 |
| chances | 0.002303382 | closed | 0.002233 | candidate | 0.002135 |
| preparedness | 0.002268227 | discussion | 0.002203 | phase | 0.002134 |
| verge | 0.002246062 | fast | 0.002183 | experts | 0.002128 |
| number | 0.002234396 | american | 0.002178 | laboratories | 0.002119 |
| stop | 0.002225956 | promising | 0.002169 | mandatoried | 0.002084 |
| future | 0.002180836 | southern | 0.00215 | reading | 0.00204 |
| jab | 0.002158177 | normal | 0.002135 | vaxxers | 0.002037 |
| national | 0.002124836 | post | 0.002133 | isn | 0.002029 |
| life | 0.002085576 | live | 0.002084 | real | 0.002029 |
| vax | 0.002083622 | team | 0.002056 | prevent | 0.002018 |
| leading | 0.002071481 | run | 0.002051 | big | 0.001996 |
| article | 0.002062384 | months | 0.002029 | public | 0.001967 |
| jointly | 0.00205521 | minister | 0.002018 | including | 0.001959 |
| prevent | 0.002048757 | latest | 0.001972 | rush | 0.001954 |
| stays | 0.002031565 | ready | 0.001968 | questions | 0.001952 |
