# Supplementary material for: Tweet Topics and Sentiments Relating to COVID-19 Vaccination Among Australian Twitter Users: Machine Learning Analysis
Source: J Med Internet Res. 2021 May 19;23(5):e26953. doi: 10.2196/26953 (PMC8136408; doi:10.2196/26953)
Supplement: Multimedia Appendix 8 [file jmir_v23i5e26953_app8.docx]

**Multimedia Appendix 8.** Top 20 probability (theta) distributions of topic 1 in tweet samples.

| Tweet samples in topic 1 | Theta |
| --- | --- |
| Idk what to believe I see reports on covid 19, &amp; the mark of the beast being a world leader &amp; how there is a vaccine or something that's going to have a microchip or a mark of the beast something to do with buy and sell and if you except the mark of the beast you will go to hell | 0.368395 |
| Will take a vaccine to best control COVID-19. The choices are about how to control rate of infection, number infected at any point and consequent deaths, at what overall cost while vaccine is being developed. People are dying. More people will die. | 0.367454 |
| Of more interest is the CDC’s 6% clarification re Covid. It means only 30 people have died in Victoria from Covid alone in seven months-which compared to annual Flu is invisible. What is Albo’s view on Covid Communism threatening enforced vaccination and fraudulent Lockdowns? | 0.366136 |
| Johnson &amp; Johnson don't know if a trial subject was receiving the vaccine or a placebo? THIS IS VERY WORRYING! J&amp;J pauses Covid vaccine trial over participant's 'unexplained illness' | 0.365767 |
| I obtained my MBChB in 2009 and postgraduate medical qualifications since. So I don’t really care about what some no mark Walter Mitty character who pretends to be a doctor thinks about my qualifications. Anyone who thinks we don’t need a Covid vax is clearly not a doctor. | 0.364298 |
| Comparing coronaviruses to the common cold is like saying a great white shark is the same as a wobbegong. Car accidents kill people, but we also have speed restrictions, traffic controls and breath testing. No current vaccine for COVID and limitations in intensive care. | 0.362243 |
| If only Morrison put as much effort into aged care as into marketing a vaccine deal...Scott Morrison's team is masterful at spin and marketing but won't even accept responsibility for the aged care crisis, let alone address it. | 0.361927 |
| It's interesting, Melbourne will have front ended 5.5 months of lockdown in the first 7 months of the pandemic hoping we will have a "covid normal" economy until a vaccine arrives. If that vaccine is available before June next year or a third wave occurs, we made the wrong choice | 0.361399 |
| An estimated 56k people die from the flu each year. Corona has killed 259 people. 3000 die from malnutrition, 3500 die from ashtma and 3000 die from peptic ulcers. ..why don't we have scare campaigns over those. No vaccine to sell of course. Corona is a huge money spinner | 0.361021 |
| I agree. I won't be getting the covid-19 vaccine jab either, I get nasty backlash from these jabs. Had a hole in my arm from one, and the meat died were they jabbed me. It fell out, and the jab did not take. They signed off as my body rejected the jab they gave me. | 0.360721 |
| Johnson &amp; Johnson pauses COVID-19 vaccine trial after participant experiences unexplained illness Remember AIDS/HIV? Vaccine has still not been devoted, this is from what I've read in Scientific Journals to be expected. | 0.36071 |
| I’ve just wasted time engaging with a nutter spewing the same crap.....Says no one dies of COVID-19, but they die of other pathology (diabetes, heart disease etc). I’m mean FFS.... imagine what it will be like when the vaccination argument starts. | 0.36045 |
| Markets down - Covid 19 concerns Markets up - Covid 19 worry fading Markets down - Covid 19 re emerging Markets up - Covid 19 not a problem Markets down - Covid 19 depression Markets up - Covid 19 vaccine Ad infinitum | 0.359223 |
| The whole point of the app is to allow some relaxation of social distancing restrictions. The alternative is to wait for a vaccine or a prep noting that a successful vaccine has never been able to be developed for other corona viruses. | 0.358991 |
| Tracking the global #COVID19 outbreak ?? Johnson &amp; Johnson pauses Covid vaccine trial ?? U.S. virus death rate is world’s worst, according to study ?? Europe tightens restrictions to regain | 0.358449 |
| What games do you want to see pros, celebrities, influencers and athletes play for charity? Some publishers won't let us use their games. We're trying to raise money for COVID-19 vaccine clinical trials. | 0.358241 |
| Could media orgs give journalists covering COVID-19 story a quick 101 on clinical trials/research process please? After 30+ years of “breakthrough” stories about HIV treatment/vaccine/cure there are some givens: - every hypothesis that proceeds to an actual trial has promise /2 | 0.358075 |
| Great choice ?? ! Why bother with medical research for a #vaccine or investing in addressing the human caused environmental degradation that is leading to #pandemics? Missiles are the answer! | 0.357925 |
| Climate change could kill billions of humans, kill much life on earth and destroy civilization and the World is not doing nearly enough to avoid this. Climate change is linked to the increased chance of pandemics. A vaccine will be found for Covid 19 in 12-18 mths. | 0.357114 |
| Should scientists develop a vaccine for Covid-19 and the world return to some sort of normality, the climate crisis will still be here. And if a vaccine is not developed, the climate crisis will still be here. | 0.357073 |
