# Supplementary material for: Tweet Topics and Sentiments Relating to COVID-19 Vaccination Among Australian Twitter Users: Machine Learning Analysis
Source: J Med Internet Res. 2021 May 19;23(5):e26953. doi: 10.2196/26953 (PMC8136408; doi:10.2196/26953)
Supplement: Multimedia Appendix 9 [file jmir_v23i5e26953_app9.docx]

**Multimedia Appendix 9.** Top 20 probability (theta) distributions of topic 2 in tweet samples.

| Tweet samples in topic 2 | Theta |
| --- | --- |
| Dotty be glad that big pharma are liability adverse. They have already stated there is no way they will agree to fast track ANY vaccine for COVID-19 without it going through every single phase safety and efficacy. Your welcome. Big pharma protects your life | 0.397601 |
| COVID-19 deaths had nothing to do with the flu vax. I’ve had the flu vax in Melbourne hotspot cases going up. Still here and kicking your lies in th teeth. | 0.390174 |
| The US has blown past the 1968 deaths (in *3 months* not a year) will shortly exceed the 1958 pandemic and is closing in on the yearly death toll from the 2009 pandemic, which included masks, quarantine, trace and track AND had antivirals and a vaccine. COVID-19 has none of these | 0.384373 |
| People die of influenza, and we do our level best with prophylactic drugs, vaccination campaigns and public health campaigns to keep that number as low as possible. More people have died in a *month* from COVID-19 than in a year from the worst flu season this century | 0.383101 |
| Not without a vaccine. If that what you are holding your breath for you are going to run out of oxygen before COVID-19 runs out of victims | 0.381387 |
| COVID-19 is now the third largest cause of death in the US. As for the non vaccine preventable diseases, there is enormous effort going into malaria prevention and treatment, maternal health and all the others | 0.381313 |
| Please explain healthy young athletes with no underlying conditions who have died from COVID-19. Herd immunity is not a belief "the level of vaccination needed to achieve herd immunity varies by disease" | 0.380459 |
| We have vaccines for MERS and animal Corona viruses so a vaccine is possible. and cornona virus doesn't mutate as fast as influenza, so we will have fewer seasonal issues. | 0.380399 |
| Thank you for explaining why vaccination, and wearing of masks to help prevent the spread of COVID-19 are so important. | 0.379041 |
| 120,000 in a year even with vaccines, masks and antivirals. COVID-19 has killed 150,000+ in just under 6 months, and we have no vaccine, might have 1 antiviral and have to rely on masks and social distancing. | 0.378963 |
| We have a MERS vaccine, so a COVID vaccine is possible. Not a certainty, but possible. We may also have working antivirals by then (not remesdivir though) | 0.374721 |
| Speaking of misinformation flu shots do not increase covid-19 infection rates. It’s just more propaganda from anti vaxxers. In fact the opposite is true it can help public health | 0.37241 |
| You filed a ludicrous request for information so narrowly designed as to not find any results, and lodged it in Canada when most of the COVID-19 vaccine research is being done elsewhere. You didn’t want to find anything. | 0.369804 |
| Entirely different corona viruses, the handful of corona viruses in seasonal colds are not SARS-COV-2 (but the point was if you develop anti bodys to SARS-COV-2 then you can make a vaccine) . | 0.368596 |
| We don’t have a Covid-19 vaccine yet. And I would like you to think about the consequences of COVID-19 seeping thoug a cancer ward. These people are some of the most vulnerable to COVID-19 | 0.368316 |
| In Australia we have had basically no flu this flu season (still rhinovirus though) as a result of the COVID-19 percussions on top of our vaccination program. Masking when you are sick, as in many Asian countries, and not “soldiering on” when you are sick, would be good. | 0.367415 |
| 1. Debunked in a trial you don’t know who gets the placebo and how got the vaccine hence why the trial was suspended until safe 2. Stroke and COVID-19 together. Many stroke patients live after a stroke. How’s your blood pressure. What is kd reference. | 0.366306 |
| In the absence of vaccination, to have immunity you need to get the disease first, that’s how immunity works. For something like COVID-19 where humans have never been exposed to it before, you are in a high risk group and likely to be severely affected. | 0.364794 |
| Alzheimer's disease was first described in 1906 (before that it was just lumped in with all the other dementias). The first effective flu vaccine was available to civilians in 1946. the 1968 pandemic killed around 100,00 US folks in over a year, COVID-19 has killed more in months | 0.364644 |
| COVID-19 is now killing more than malaria and tuberculosis combined, it is a million *extra* deaths that could have been prevented. As I type, malaria and tuberculosis campaigns are on going, as well as vaccination for vaccine preventable diseases. We are working on it. | 0.363061 |
