# Supplementary material for: Tweet Topics and Sentiments Relating to COVID-19 Vaccination Among Australian Twitter Users: Machine Learning Analysis
Source: J Med Internet Res. 2021 May 19;23(5):e26953. doi: 10.2196/26953 (PMC8136408; doi:10.2196/26953)
Supplement: Multimedia Appendix 10 [file jmir_v23i5e26953_app10.docx]

**Multimedia Appendix 10.** Top 20 probability (theta) distributions of topic 3 in tweet samples.

| Tweet samples in topic 3 | Theta |
| --- | --- |
| But there is no vaccination for either strain of COVID-19 yet. Flu shot doesn't protect against it either. Vaccinations are also not cures, nor can they prevent a person carrying or catching thing they protect you from. I'm NOT an anti-vaxxer BTW, just not an anti anti-vaxxer.?? | 0.372743 |
| world needs a much faster test (dare I say they are working on it) Perhaps even with a vaccine we many need a real time test for COVID. Maybe a condition of entry for the next phase of living with this virus - a test that instantaneously give you a result. ?? | 0.366039 |
| Y we been in lockdown is the Q? Proof NO vaccine is needed. ANy 1 getting Flu shot should B asking 4 whats in the flu shot, Legally Doctors not allowed 2 refuse u might b surprised 2 find sum dead Covid strins in their | 0.365918 |
| What’s the option? Watch it go down, then one case emerges and we all lockdown for another 400 days? All the ppl touting a long lockdown are on govt pay. I am not and have 7 employees, business dead. There is no cure, and no vaccine. Still not one for the cold which is corona. | 0.363687 |
| HCQ in harness with Zinc and Azithromycin-administered early and in normal doses, for five days-has a 99.3% cure rate for Covid. Tests not employing these methods are Politicised and designed to fail in order to validate its banning and completely unnecessary vaccine protocols. | 0.361736 |
| We have got it all wrong, we’re so focussed on the COVID virus, we are not addressing the root cause. The biggest issue you have atm is ‘Testing’. More testing leads to more COVID cases. Stop the test, stop the disease. We don’t need a COVID vaccine, we need a ‘Testing’ vaccine. | 0.359997 |
| HOA (Heads of Agreement) to secure COVID-19 VACCINE for AUSTRALIANS now established. Thats the business method to go! Final tests to follow good results in multi tests completed. Oxford University team. Quality expected as already indicated! | 0.359732 |
| if the vaccine for covid is estimated as possible for q4 2020/q1 2021) is it reasonable to assume we are now confident that there is a reilable antibody test for Covid-19 ? If so, would antibody testing now help to understand underlying population immunity? | 0.359553 |
| Viruses can cause pneumonitis (lung inflammation) and/or be complicated by superadded bacterial pneumonia, which can be due to a wide variety of bacteria. No harm and good to have the vaccine generally, but unlikely to prevent COVID-related pneumonia on any significant scale | 0.359531 |
| This kinda makes sense. Anti vaxxers refuse covid vaccine. Kids catch it but are symptom-free. Kids bring it home. Anti vaxxers get covid-19 and die. Karma. | 0.359503 |
| Google "herd immunity." It's not a new concept although anti-vaxxers like you keep trying to tear it apart so we all get to live with Polio again... Before you ask, no herd immunity doesn't apoly to Covid as a vaccine doesn't yet exist. So grow up and wear a damn mask. | 0.359334 |
| speaks of ‘the pub test’. Remember this: if he was Premier, pubs would be open, many ppl would be dead &amp; fighting for their lives. He believes in herd immunity without a vaccine - in other words, genocide. Mr OBrien will never pass many tests. He’s a dud. | 0.359102 |
| Australia has created lab version of corona virus from a patient to create a vaccine to counter it. We are suggesting using stuff which has never been tested or certified to fight the virus. Good job GoI. With every passing day you are putting the countrymen in new dangers. | 0.358686 |
| Italian #anti-vaxxers aren’t backing down in the face of #COVID-19. Bizarrre given Italy’s disastrous COVID-19 experience. Guess anti-vaxxers can place some reliance on the majority trusting Science &amp; being jabbed. | 0.358308 |
| Herd immunity in Australia without vaccine 25M popn 80% to be infected = 20M per day = 55,000 14 day infection = 770k at one time 15% hospitalised for 10 days = 82,500 beds 5% ICU for 10 days = 27,500 ventilators All on top of ordinary load of system Build! | 0.358022 |
| COVID-19 is from the SARS virus family, thats why suddenly there are highly accurate Test Kits available! But, they never had a vaccine or cure for that family of viruses... SARS was Bird Flu...COVID is Bat Flu!!! Eat more Bats! | 0.357916 |
| COVID LINGO DECODED : ? “We must do mass testing” (more tests = more positives = more lockdown) ? “Vaccine breakthrough!” (vaccines take years = years of #Lockdown) ? “Masks Matter” (Visual propaganda. Fear porn = Lockdown matters) | 0.357761 |
| I really don't understand your position. We're in a Pandemic. Front line US ER drs using HCQ are saving lives. Turkey, Switz, S.Korea &amp; India's biggest slum, saved by HCQ. So you get COVID &amp; you'll reject HCQ? Bill Gates has a new beaut 'tested' vaccine just for you. | 0.357261 |
| I assume PM &amp; CMO won't talk about herd immunity again, but it is code for something very scary not suitable for a six year old. Before a vaccine is available it assumes people who recover from Covid-19 have long term immunity, reducing spread. Those who don't recover are dead. | 0.357031 |
| Of course they won't just give it away but neither are they making a profit and 300,000 doses is less than 0.5% of US population (each person needs 2 doses). Europe to get 400 million doses. Astrazeneca have signed deals with many countries. | 0.356925 |
